# Novel ^1^H/^19^F double‐tuned coil using an asymmetrical butterfly coil

**DOI:** 10.1002/mp.17890

**Published:** 2025-05-19

**Authors:** Suk‐Min Hong, Chang‐Hoon Choi, Jörg Felder, N. Jon Shah

**Affiliations:** ^1^ Institute of Neuroscience and Medicine 4 INM‐4 Forschungszentrum Jülich Jülich Germany; ^2^ Institute of Neuroscience and Medicine 11 INM‐11, JARA Forschungszentrum Jülich Jülich Germany; ^3^ JARA ‐ BRAIN ‐ Translational Medicine Aachen Germany; ^4^ Department of Neurology RWTH Aachen University Aachen Germany

**Keywords:** MRI, RF coil, 19F

## Abstract

**Background**: Fluorine‐19 (^19^F) magnetic resonance imaging (MRI) is a non‐invasive imaging tool for the targeted application of fluorinated agents, such as cell tracking, and for the demonstration of oximetry. However, as the SNR of ^19^F is significantly weaker than that of proton (^1^H) imaging, the ^19^F coil must be combined with ^1^H coils for anatomical co‐registration and B_0_ shimming. This is difficult due to the strong coupling between the coils when they are in proximity, and is problematic since the Larmor frequency of ^19^F is 94% that of ^1^H, further increasing the potential for coupling between the ^1^H and ^19^F elements.

**Purpose**: Conventional double‐tuned coil methods tend to generate loss compared to single‐tuned reference coils. The asymmetrical butterfly coil has a split resonance peak, which can cover frequencies of ^1^H and ^19^F without losses arising from lossy traps or switching circuits. In this study, the use of an asymmetrical butterfly coil was evaluated for ^1^H/^19^F applications.

**Methods**: To increase quadrature efficiency at both the ^1^H and ^19^F frequencies, the left and right loops of the butterfly coil were tuned asymmetrically. The coil's tuning and performance were evaluated in simulations and MR measurements, and the results were compared to a dimension‐matched single‐tuned loop coil.

**Results**: The split resonance peak of the asymmetrical butterfly coil successfully spanned the ^19^F to ^1^H frequency. It operated with higher quadrature efficiency at both ^1^H and ^19^F frequencies and demonstrated superior receive sensitivity and SNR compared to the dimension‐matched single‐tuned loop coil.

**Conclusions**: The split resonance peak of the asymmetrical butterfly coil supported both ^1^H and ^19^F frequencies, delivering a higher SNR than that of the single‐tuned loop coil. Since the asymmetrical butterfly coil can cover ¹H and ¹⁹F frequencies without loss and provides higher efficiency than the reference single‐tuned coil, it can be effectively utilized for ¹H/¹⁹F MRI applications.

## 1 INTRODUCTION

Magnetic resonance imaging (MRI) is a medical imaging tool that uses radiofrequency signals to excite nuclear spins within a strong static magnetic field, subsequently detecting the echo signal to reconstruct image data.^1^ The primary nucleus targeted in MRI has been the proton (^1^H) due to its high relative sensitivity and concentration in the human body.^2^ Proton‐based MRI has provided detailed anatomical information and has enabled advanced methods for assessing brain function, such as functional MRI (fMRI)^3^ and brain connectivity.^4^ As MRI imaging techniques and technology have developed, interest in imaging using other nuclei, so‐called X‐nuclei, has also grown. X‐nuclei imaging is particularly useful as it can provide complementary information to that provided by ^1^H. For example, phosphorus‐31 imaging can be used to evaluate energy metabolism,^5^ while sodium‐23 imaging can be used in brain tumor classification.^6^

Fluorine‐19 (^19^F) MRI has 84% of the relative sensitivity of ^1^H and generates no background signal from body tissue, making it suitable for visualizing fluorinated targets.^7^, ^8^ ^19^F nanoprobes have been evaluated in the context of cell tracking and labelling,^9^, ^10^ and ^19^F MRI can be used to demonstrate oximetry in the brain, kidneys, and placenta.^11^, ^12^, ^13^, ^14^ However, the SNR of ^19^F is very weak compared to that of ^1^H. Therefore, the ^19^F coil must be used alongside ^1^H coils for B_0_ shimming and co‐registration. Various methods exist for supporting multiple nuclei frequencies, including the use of traps with separate coils, geometries for double‐resonance and PIN diode switching.^15^, ^16^, ^17^ The Larmor frequency of ^19^F is 94% that of ^1^H, making them very close to each other. In this condition, recent studies show that the use of PIN diode switching provides minimum loss at ^19^F frequency.^15^

To acquire the MR signal, both transmission and reception processes are required. The B_1_^+^ and B_1_^−^ fields are circularly polarized components involved in transmission and reception, respectively.^18^ Multi‐channel receive‐only coils are commonly used to increase SNR and minimize acquisition time with parallel imaging techniques.^19^, ^20^ Furthermore, they have also been evaluated for use with ^19^F MRI.^21^ Multi‐channel receive‐only array coils typically consist of linearly polarized (LP) mode coils, which generate both B_1_^+^ and B_1_^−^ fields with equal magnitudes.^22^ To enhance SNR, 2‐channel quadrature surface coils can be employed.^23^, ^24^, ^25^, ^26^ The quadrature surface coil offers higher transmit efficiency and receive sensitivity due to its higher quadrature efficiency compared to conventional loop coils. Higher quadrature efficiency results in a more focused B_1_^+^ distribution during the transmission phase, whilst a higher B_1_^−^ field strength compared to B_1_^+^ in the receive phase. A recent study has evaluated the use of a single‐channel quadrature mode butterfly coil, which generates higher quadrature efficiency than a size‐matched loop coil.^27^ The quadrature operation mode of a 2‐channel surface coil involves feeds with identical voltage but a 90‐degree phase difference between the two channels. To operate the single‐channel asymmetrical butterfly coil in quadrature mode, the coil was tuned with asymmetrical capacitance values, resulting in a split resonance peak. The asymmetrical butterfly coil was found to provide a higher SNR than a size‐matched loop coil.^27^

In this study, an asymmetrical butterfly coil was evaluated for the ^1^H/^19^F applications at 7T MRI. The coil was tuned to cover both the ^1^H and ^19^F frequencies at 7T MRI, and its tuning and performance were evaluated using simulations and MR measurements. The results were compared to a conventional loop coil.

## 2 METHODS

### 2.1 Simulation

**FIGURE 1:**
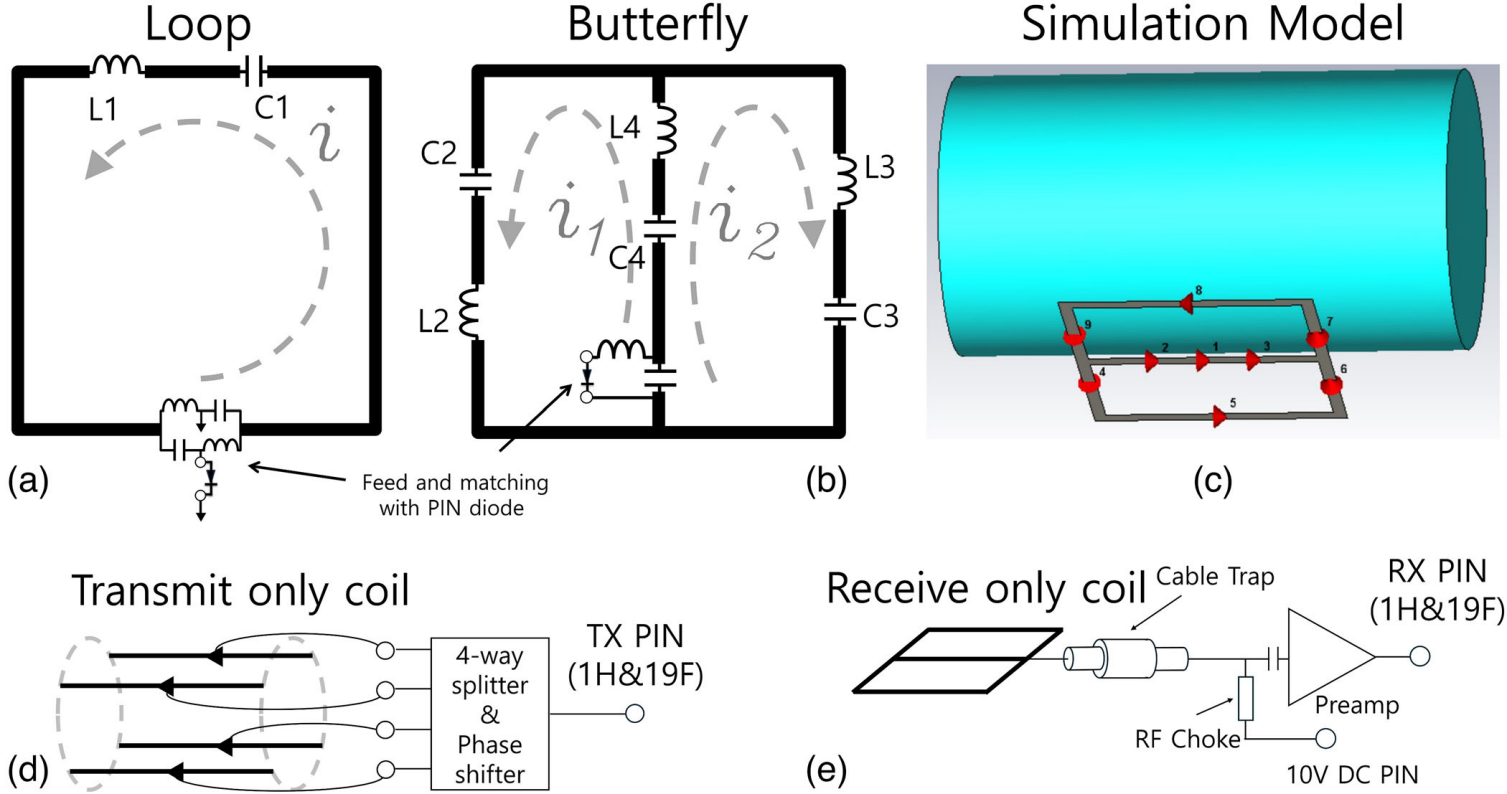
Schematic of the loop (a) and butterfly coils (b), the CST simulation model of a 10 × 10 cm^2^ butterfly coil (c) and the interface of transmit‐only coil (d) and receive‐only coil (e).

To evaluate the tuning and receive sensitivity of the asymmetrical butterfly coil at both ^1^H and ^19^F frequencies (300 and 282 MHz) for 7T MRI, a FIT simulation was conducted using the CST Microwave Studio (Dassualt Systems, Vélizy‐Villacoublay, France). Figure 1 shows the schematics of the butterfly and loop coils along with the corresponding CST simulation models and the system interface of the transmission‐only coil and the receive‐only coil. The asymmetrical butterfly coil consists of left and right loops, with the feed located on the center conductor. The tuning of the RF coil is based on the LC resonance tuning. The loop coil (Figure 1a) is tuned by adjusting the capacitance value, C1, which cancels the inductance, L1. For the butterfly coil (Figure 1b), the current is split and combined again to feed. The left and right loops are tuned by adjusting the capacitance values of C2 and C3. Conventionally, the left and right loops of the butterfly coil are tuned with symmetrical capacitance values, causing the current to split with the same phase and magnitude but in opposite directions, resulting in LP mode. A recent study evaluated the asymmetrical tuning of a butterfly coil, which can provide asymmetrical impedance and phase delay of the current between the left and right loops.^27^ This phase delay between the loops generates higher quadrature efficiency and increases the signal‐to‐noise ratio (SNR). In this simulation, capacitors of 3 × 6.5 pF and 3 × 8.4 pF were used for the left and right loops of the asymmetrical butterfly coil.

The 10 × 10 cm^2^ loop coil, the butterfly coil in LP mode and the asymmetrical butterfly coil were loaded with a cylindrical phantom (diameter = 11 cm, permittivity = 80, and conductivity = 1.0 S/m). The 50 Ω ports were placed at the capacitor and feed locations. After 3D simulations, all 50 Ω ports were replaced with capacitors, inductors, and feeds to evaluate tuning in co‐simulation.^28^ Based on the tuned s‐parameters at 7T ^1^H and ^19^F frequencies, the 3D electric and magnetic field data were combined. The receive sensitivity of the conventional loop coil and asymmetrical butterfly coil were calculated by dividing the B_1_^−^ field by the square root of the absorbed power. The transmission efficiency was also calculated by dividing the B_1_^+^ field by the accepted power. The B_1_^−^ and B_1_^+^ fields were determined by combining the B_x_ and B_y_ fields.^18^ The mean transmission efficiency and receive sensitivity were then calculated within the phantom volumes. The quadrature efficiency was calculated by dividing the B_1_^−^ field distribution by the sum of B_1_^+^ and B_1_^−^.^24^ *^,^* ^26^

### 2.2 MR measurement

The 10 × 10 cm^2^ single‐tuned loop and 10 × 10 cm^2^ asymmetrical butterfly coil were constructed using copper tape (3 M, St. Paul, Minnesota, USA) and tuned using non‐magnetic capacitors (Dalicap Tech. Co. Ltd., China) based on the simulation results. To operate the loop and butterfly coils in receive‐only mode, PIN diodes were incorporated in the feed circuits (Figure 1a and Figure 1b), and the ports were connected to a preamp. The noise figures of the preamp at the ^1^H and ^19^F frequencies were 0.8 dB and 0.9 dB, respectively. The PIN diodes were controlled with a 10 V DC PIN (Figure 1e). When the PIN diode is in the active condition, the feed circuits form parallel resonance, resulting in a receive‐only coil in a detuned state. The loop coil and the butterfly coils were loaded with a 2 L bottle phantom (diameter = 11 cm, length = 20 cm) containing KH_2_PO_4_ 30 mM + NaF 50 mM. The bottle phantom was driven by a 4‐channel dipole antenna transmission‐only array coil at both ^1^H and ^19^F frequencies (Figure 1d).

All MR measurements were conducted on a Terra 7T MRI scanner (Siemens Healthineers, Erlangen, Germany). The 7T MRI scanner supports ^1^H and ^19^F operation at 297.18 and 279.578 MHz. All constructed coils were tuned to support these scanner frequencies. To evaluate the SNR of the asymmetrical butterfly coil, 2D gradient echo images were acquired at both the ^19^F frequency (TR = 500 ms, TE = 3.02 ms, flip angle = 90°, pixel band width = 250 Hz, average = 16, matrix size = 64 × 64, pixel size = 3 × 3 mm^2^, and slice thickness = 10 mm) and the ^1^H frequency (TR = 500 ms, TE = 3.17 ms, flip angle = 90°, pixel band width = 250 Hz, matrix size = 256 × 256, pixel size = 0.75 × 0.75 mm^2^, and slice thickness = 3 mm). Noise was evaluated by acquiring images again with 0 V excitation. The SNR map was calculated by dividing the pixel values by the standard deviation of the image at 0 V.

## 3 RESULTS

**FIGURE 2:**
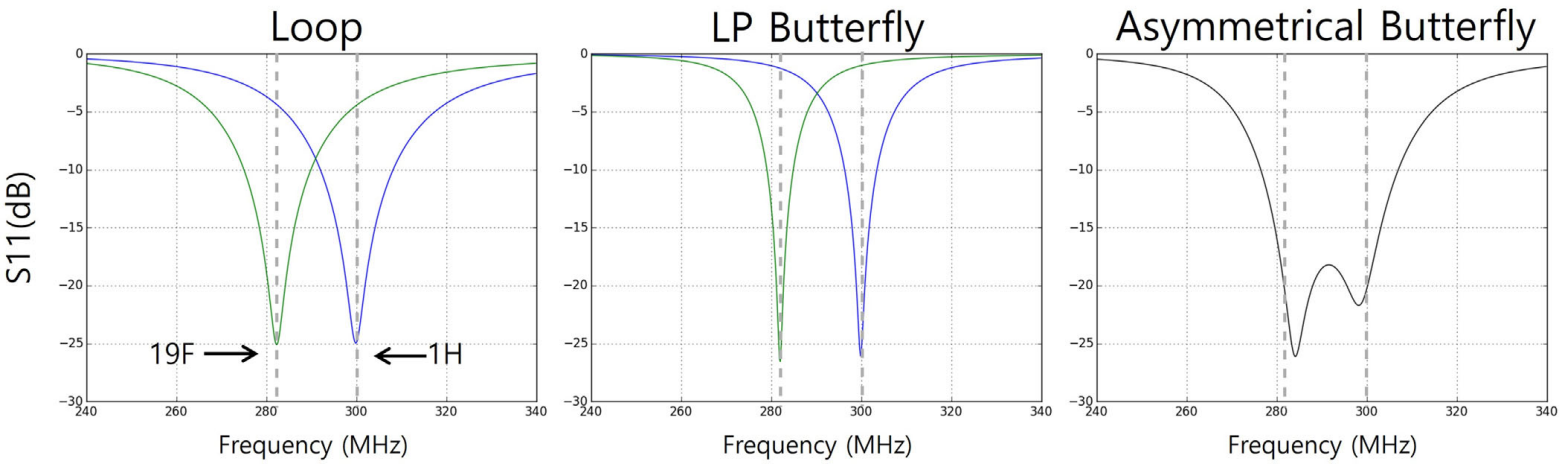
S‐parameter of the single‐tuned loop coil, LP butterfly coil, and asymmetrical butterfly coil. The green and blue lines indicate that the coils were tuned at the ^19^F and ^1^H frequencies, respectively. The dashed lines indicate the location of the ^19^F (282 MHz) and ^1^H (300 MHz) frequencies.

Figure 2 shows simulated reflection coefficients of the loop coil, the LP mode, and the asymmetrical butterfly coil. Table 1 displays the S11 values under different RF coil tuning conditions. The single‐tuned loop coil and LP butterfly coil provided limited S11 coverage values and could not simultaneously cover the ^1^H and ^19^F frequencies. When these coils were tuned for either ^1^H or ^19^F frequencies, they produced suboptimal reflection at the opposite frequency. In contrast, the asymmetrical butterfly coil shows split resonance frequencies, covering both the ^1^H and ^19^F frequencies, with values better than – 20 dB at both frequencies.

**TABLE 1:** Simulated S11 of a single‐tuned loop coil, an LP butterfly coil and an asymmetrical butterfly coil.

		^19^F frequency (dB)	^1^H frequency (dB)
loop	19F tuned	−25.0	−4.5
	1H tuned	−4.3	−25.0
LP butterfly	19F tuned	−26.5	−1.0
	1H tuned	−1.3	−25.7
Asymmetrical butterfly		−21.2	−20.1

*Note*: The S11 values are shown in Figure 2 at the^1^H and ^19^F frequencies.

**FIGURE 3:**
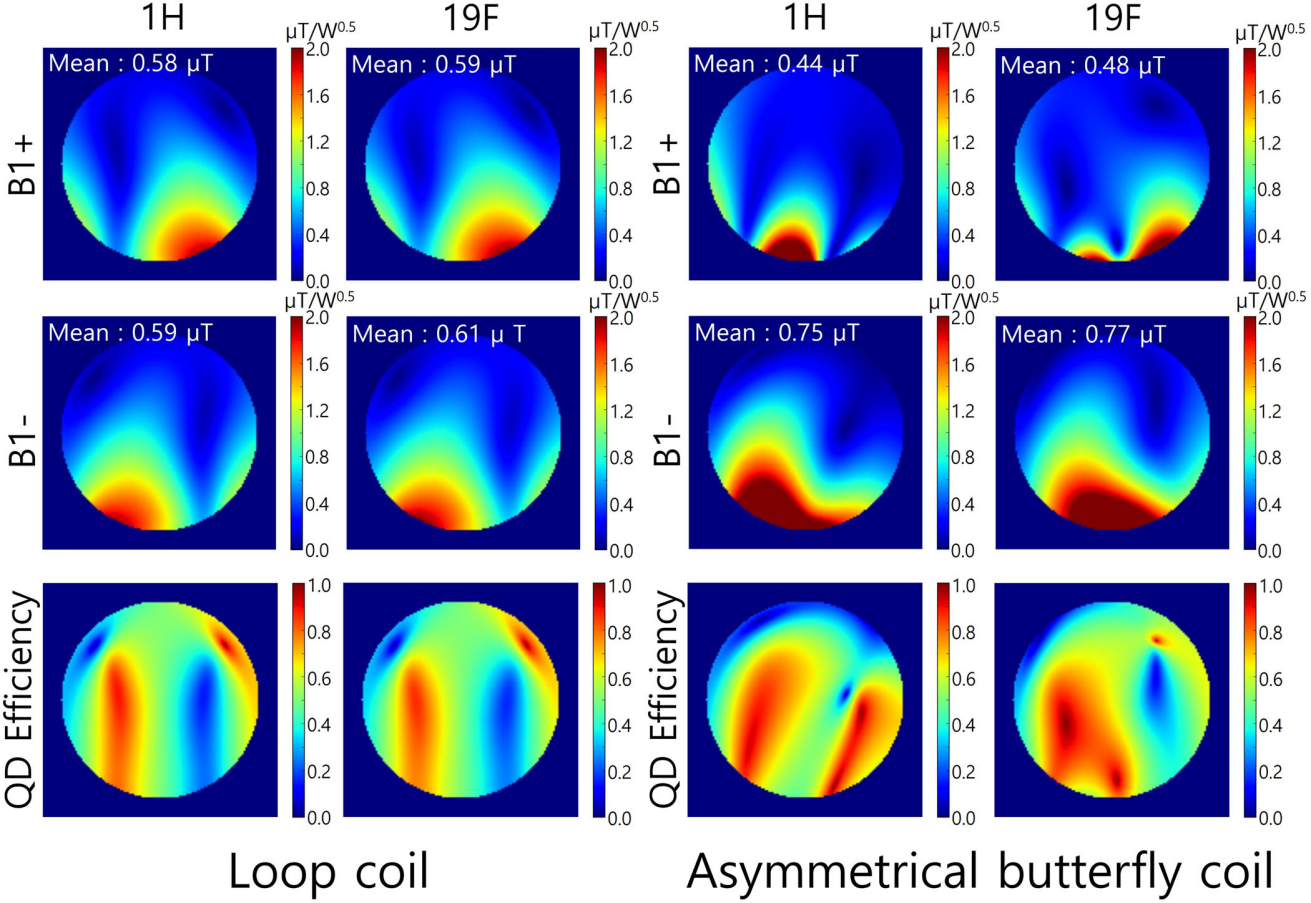
Simulated transmit efficiency, receive sensitivity, and quadrature (QD) efficiency of the single‐tuned loop coil and asymmetrical butterfly coil at the ^1^H and ^19^F frequencies, respectively.

Figure 3 shows the simulated transmission efficiency, receive sensitivity and quadrature efficiency of the single‐tuned loop coil and asymmetrical butterfly coil at ^1^H and ^19^F frequencies. The single‐tuned loop coil provided similar averaged values of transmission efficiency and receive sensitivity, with mirrored patterns in the left and right directions for both frequencies. In contrast, the asymmetrical butterfly coil exhibited higher receive sensitivity than transmission efficiency, with mean receive sensitivity values exceeding those of the loop coil at both ^1^H and ^19^F frequencies. The receive sensitivity patterns of the asymmetrical butterfly differed slightly between the ^1^H and ^19^F frequencies. The asymmetrical butterfly coil provided higher quadrature efficiency compared to the conventional loop coil at both ^1^H and ^19^F frequencies. However, the higher quadrature efficiency pattern of the butterfly coil was not symmetrical or uniform.

**FIGURE 4:**
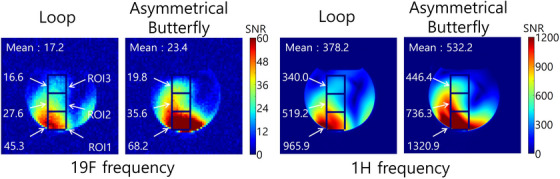
Measured SNR maps of the single‐tuned loop coil and asymmetrical butterfly coil at the ^19^F and ^1^H frequencies. The square boxes show the locations of the ROIs. The mean values were calculated in the whole phantom area.

Figure 4 shows measured SNR maps of the single‐tuned loop coil and asymmetrical butterfly coil at both ^1^H and ^19^F frequencies. The asymmetrical butterfly coil provided a higher averaged SNR than the single‐tuned loop coil at both frequencies. The SNR values in the ROIs indicate that the asymmetrical butterfly coil offered higher averaged SNR values as well as higher SNR over the whole coil coverage. The measured results were consistent with the simulated receive sensitivity.

## 4 DISCUSSION AND CONCLUSIONS

The simulation results showed that the single‐tuned loop coil provided identical B_1_^+^ and B_1_^−^ averaged field strength, therefore indicating that they operate in LP mode. In contrast, the asymmetrical butterfly coil provided higher B_1_^−^ field strength compared to its B_1_^+^ field. While the total sum of B_1_^+^ and B_1_^−^ fields provided by the single‐tuned coil and asymmetrical butterfly coil are almost identical, with a difference of less than 5%, the asymmetrical butterfly coil provided more focus on the B_1_^−^ field. The asymmetrical butterfly coil provided higher quadrature efficiency compared to the loop coil at both ^1^H and ^19^F frequencies. Although the quadrature efficiency patterns of the asymmetrical butterfly coil were not uniform, the higher quadrature efficiency values of the butterfly resulted in higher averaged B_1_^−^ field strength compared to the loop coil. The supporting information (Video S1) shows the quadrature efficiency and H‐field vector depending on the time phase at ^19^F frequency. In the area with high quadrature efficiency, the H‐field vectors can be seen to be rotating in a clockwise direction. This aligns with the MR measurements, which also showed that the asymmetrical butterfly coil produced a higher SNR than the single‐tuned loop coil.

The tuning capacitor values for the asymmetrical butterfly coil were selected to generate a more focused B_1_^−^ field distribution than B_1_^+^. In principle, the use of the asymmetrical butterfly coil during the transmission phase would be possible by changing the tuning capacitor values on the left and right loops or by flipping the coil's orientation. This process is identical to that used to select the polarity for 2‐channel quadrature surface coils. However, tuning the asymmetrical butterfly coil for optimal performance at both the ^1^H and ^19^F frequencies would be limited when it is used for the transmit coil. Due to local SAR, the subject needs to be placed at a distance from the transmit coils compared to the receive‐only coil. In this condition, the coil tuning is very sensitive to the loading, and the use of a single matching capacitor (Figure 1b) would be insufficient to cover both ^1^H and ^19^F frequencies.

Some studies evaluated the use of PIN diode switching for ^1^H/^19^F applications, taking advantage of the close Larmor frequencies of ^1^H and ^19^F. The PIN diode switching concept provided minimum loss at X‐nuclei frequencies compared to single‐tuned coils. However, the use of additional components, such as PIN diodes,^15^ trap circuits, and MEMs^16^, generates loss; therefore, the goal or reference of the double‐tuned coil is geometrically identical to a single‐tuned coil. In contrast, the asymmetrical butterfly coil does not require any additional components, as its left and right loops are tuned asymmetrically to generate a split resonance. The MR measurement results at both the ^1^H and ^19^F frequencies were in agreement with those of the simulation, demonstrating that the asymmetrical butterfly coil provided higher SNR compared to the size‐matched single‐tuned loop coil at both frequencies.

The asymmetrical butterfly coil provided higher sensitivity and deeper penetration compared to the conventional loop coil. To cover the whole brain or another object, a multi‐channel phased array design is required. For the conventional phased array design, several decoupling methods can be used, such as adjusting the overlapping distance,^19^ preamp decoupling,^19^, ^29^ inductive decoupling,^30^, ^31^ and self‐decoupling element.^32^ These decoupling methods could also be employed in the phased array design with an asymmetrical butterfly coil. However, preamp decoupling provides a narrower bandwidth compared to the frequency difference between the ^1^H and ^19^F frequencies. The use of preamp decoupling for the asymmetrical butterfly coil array should be evaluated and compared with 50 Ω termination in further studies.

The asymmetrical tuning of the left and right loops of the butterfly coil results in a split resonance peak that covers both ^19^F and ^1^H frequencies. In simulations, the asymmetrical butterfly coil demonstrated higher quadrature efficiency and receive sensitivity than the loop coil at both ^1^H and ^19^F frequencies. The measurement results confirmed these findings. Since the asymmetrical butterfly coil covers both the ¹H and ¹⁹F frequencies without any lossy double‐tuning methods while additionally providing a higher SNR compared to a size‐matched single‐tuned loop coil, it can be effectively utilized for ¹H/¹⁹F MRI applications at ultra‐high fields.

## CONFLICT OF INTEREST STATEMENT

The authors declare no conflicts of interest.

## Supporting information



Supporting Information (Video S1)
